# An Open-Label Case Series of Glutathione Use for Symptomatic Management in Children with Autism Spectrum Disorder

**DOI:** 10.3390/medsci11040073

**Published:** 2023-11-15

**Authors:** Karam Radwan, Gary Wu, Kamilah Banks-Word, Ryan Rosenberger

**Affiliations:** 1 Department of Psychiatry & Behavioral Neuroscience, University of Chicago Medical Center, Chicago, IL 60637, USAryan.rosenberger@aah.org (R.R.); 2 Department of Psychiatry & Behavioral Sciences, Rosalind Franklin University, North Chicago, IL 60064, USA; gary.wu@rosalindfranklin.edu

**Keywords:** oxidative stress in autism, glutathione, transsulfuration pathway, autism

## Abstract

**Simple Summary:**

Autism spectrum disorder (ASD) is a neurodevelopmental disorder that can cause impaired social–emotional interactions, impaired language and communication skills, repetitive or restrictive behaviors, and sometimes aggressive behavior. An emerging topic in research is the imbalance between bodily oxidative systems and anti-oxidative stress in autism spectrum cases. Glutathione (an antioxidant agent) is involved in many anti-oxidative stress systems, but our research target is to study the role of glutathione as a neuro-protective agent. Our pilot study demonstrates general glutathione tolerability and some efficacy in decreasing problematic behaviors observed in children with ASD.

**Abstract:**

Autism spectrum disorder (ASD) is a type of neurodevelopmental disorder that has been diagnosed in an increasing number of children around the world. The existing data suggest that early diagnosis and intervention can improve ASD outcomes. The causes of ASD remain complex and unclear, and there are currently no clinical biomarkers for autism spectrum disorder. There is an increasing recognition that ASD might be associated with oxidative stress through several mechanisms including abnormal metabolism (lipid peroxidation) and the toxic buildup of reactive oxygen species (ROS). Glutathione acts as an antioxidant, a free radical scavenger and a detoxifying agent. This open-label pilot study investigates the tolerability and effectiveness of oral supplementation with Opitac^TM^ gluthathione as a treatment for patients with ASD. The various aspects of glutathione Opitac^TM^ glutathione bioavailability were examined when administered by oral routes. The absorption of glutathione from the gastrointestinal tract has been recently investigated. The results of this case series suggest that oral glutathione supplementation may improve oxidative markers, but this does not necessarily translate to the observed clinical improvement of subjects with ASD. The study reports a good safety profile of glutathione use, with stomach upset reported in four out of six subjects. This article discusses the role of the gut microbiome and redox balance in ASD and notes that a high baseline oxidative burden may make some patients poor responders to glutathione supplementation. In conclusion, an imbalance in redox reactions is only one of the many factors contributing to ASD, and further studies are necessary to investigate other factors, such as impaired neurotransmission, immune dysregulation in the brain, and mitochondrial dysfunction.

## 1. Introduction

Autism spectrum disorder (ASD) is a neurodevelopmental disorder defined by the Diagnostic and Statistical Manual of Mental Disorders, fifth edition (DSM-5) as observable behavioral deficits in social communication and social interaction across multiple contexts. Oftentimes, patients with ASD present with difficulties relating to others in the form of repetitive patterns of behaviors and limited interests. Although ASD can present at any age, the disorder usually manifests early in childhood and can be reliably diagnosed by age 2 [1]. DSM-5 (Appendix A) replaced and folded the DSM-IV subtypes of Pervasive Developmental Disorder (PDD), Autistic Disorder, Asperger’s Disorder, Childhood Disintegrative Disorder under a single umbrella term of ASD. According to the most recent data from the CDC’s Autism and Developmental Disabilities Monitoring (ADDM) network published in December 2021, an average of 1 in every 44 (2.3%) 8-year-old children were estimated to have ASD in 2018 [2]. Additionally, ASD is 4.2 times as prevalent among boys (3.7%) compared to girls (0.9%) [2].

The cause of ASD is currently unknown and involves a mix of genetics and environmental factors. Based on the current literature, ASD involves many complex biological processes that manifest as physiologic and metabolic abnormalities. Namely, ASD has been reported to be in association with impaired neurotransmission, immune dysregulation in the brain, mitochondrial dysfunction, and increased oxidative stress [3,4,5].

The neurodevelopmental nature of ASD is partially explained by the pathophysiologic imbalance of neurochemical signaling in the central nervous system. ASD has been observed to be associated with changes to neurotransmission involving gamma aminobutyric acid (GABA), glutamate, serotonin, dopamine, acetylcholine, N-acetyl aspartate, and endogenous opioids, amongst others [6]. Changes to the production, degradation, and response pathways of these neurotransmitters may affect cellular functions including differentiation, migration, and apoptosis.

There is growing evidence that supports a role for immune dysregulation in the brain in ASD. Neurobiological studies on children with ASD have demonstrated an excess in neuronal synapses in the cerebral cortex. An impairment in synaptic pruning is observed to lead to social and behavioral dysregulation, as is seen in ASD. On an immunologic level, brain microglia are crucial in the synaptic refinement process [7]. Specifically, studies have shown that the loss of mTOR-dependent autophagy pathways impairs synaptic pruning which further underscores immune dysregulation as a contributor to the development of ASD [8,9].

Synaptic transmission is an energy intensive process that requires large amounts of ATP produced by mitochondria. The synthesis of proteins and dendrites for synaptic plasticity is also another energy intensive process. As such, functional mitochondria are crucial to neurodevelopment. An early study in 1985 cited lactic acidosis in a subset of children with ASD, which further paved the way to explore the association between mitochondrial dysfunction in ASD [10]. Current studies on mitochondrial dysfunction hint at enzymatic dysregulation in the tricarboxylic acid cycle and the electron transport chain [3,4]. Mitochondria also play other roles in synaptic regulation including calcium buffering, the regulation of neurotransmitter release, and reduction in oxidative stress [11]. Mitochondrial dysfunction is also noted to be associated with other ASD-like neurodevelopmental disorders including Down syndrome, DiGeorge syndrome, Rett syndrome, and tuberous sclerosis complex [11,12,13,14].

Lastly, an imbalance in oxidation–reduction (redox) reactions in cells favor pro-oxidant agents, such as free radicals, which promote oxidative stress. More specifically, the generation of ROS outweighs the body’s ability to remove them. In low concentrations, reactive oxygen species act as signaling molecules that induce apoptosis and dysregulates cell proliferation via changes to gene expression [15]. On a molecular level, ROS can destroy polyunsaturated fatty acids that make up the cell membrane. Additionally, ROS can oxidize amino acids leading to DNA strand breakage and DNA protein crosslinking resulting in mutations. ROS damage is a positive feedback system whereby more ROS are released downstream, resulting in further cellular damage. The resulting pro-oxidative state and cytotoxicity causes brain inflammation, the disruption of the blood–brain barrier, edema, and presents as the phenotypic symptoms of ASD [16,17].

In order to protect itself, the body utilizes an antioxidant defense mechanism that includes both enzymatic and non-enzymatic ways to remove ROS. Enzymatic defense includes the biological action of superoxide dismutase, catalase, peroxiredoxins, glutathione peroxidase, and several enzymes in the ascorbate-glutathione pathway [18]. Non-enzymatic defense includes vitamins (vitamins C and E), β-carotene, uric acid, and glutathione, among others [19]. An emerging topic of research is the restoring of the redox balance between bodily oxidative systems and anti-oxidative responses. The literature focuses on the hypothesis that reducing oxidative burden improves symptoms and social functioning in patients with ASD.

**Figure 1:**
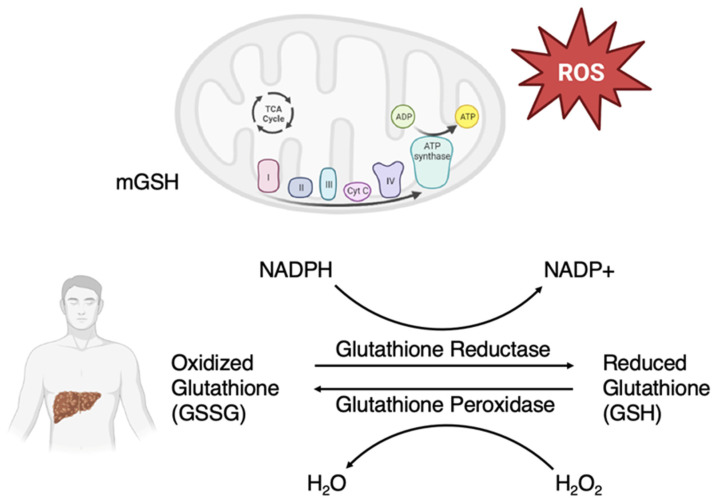
Glutathione redox cycle. Glutathione is primarily synthesized in the liver. Oxidized glutathione (GSSG) is then reduced via glutathione reductase and nicotinamide adenine dinucleotide phosphate (NADPH) into reduced glutathione (GSH). GSH is a major antioxidant in the body that primarily acts to eliminate reactive oxygen species (ROS). A major source of ROS is from oxidative phosphorylation in mitochondria. Mitochondrial GSH (mGSH) plays an important role in maintaining the redox balance in the body.

**Figure 2:**
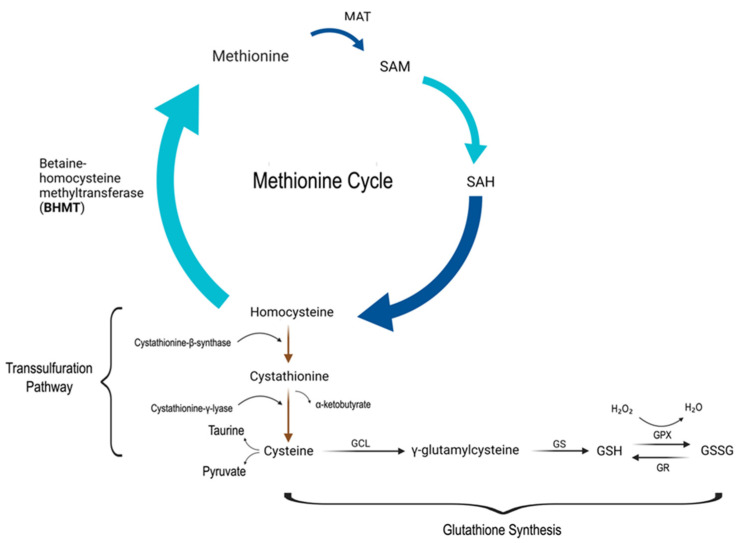
Methionine cycle, transsulfuration pathway, and production of glutathione. The top portion of the figure depicts the methionine pathway, in which methionine is catalyzed by methionine adenosyltransferase (MAT) to form S-adenosyl-methionine (SAM), which is a primary methyl donor in the body. SAM is converted to S-adenosylhomocysteine (SAH) after donating its methyl group, and later hydrolyzed to form homocysteine. Homocysteine can be remethylated to form methionine using betaine-homocysteine methyltransferase (BHMT), thus closing the methionine cycle. The transsulfuration pathway depicted starts with the condensation of homocysteine and serine using cystathionine-β-synthase (rate-limiting step) to form cystathionine. Cystathionine is further hydrolyzed by cystathionine-γ-lyase to produce cysteine, which is further used to produce taurine and pyruvate. Glutathione production starts with cysteine, which is combined with glutamate via the enzyme glutamate cysteine ligase (GCL). The resulting γ-glutamylcysteine is then converted to reduced glutathione (GSH) via glutathione synthetase (GS). GSH is used for the reduction of ROS via glutathione peroxidase (GPX) and the resulting glutathione disulfide (GSSG) can be converted back to GSH via glutathione reductase (GR).

One of the most studied antioxidants is glutathione due to its ubiquitous nature and its role as the most abundant non-protein thiol antioxidant in mammalian tissue. Glutathione is a naturally occurring cysteine-glutamate-glycine tripeptide that is synthesized primarily in the liver with significant antioxidative action. In addition to its key role in redox signaling, crucial in the detoxification of xenobiotics, glutathione is an important factor in regulating cell proliferation, apoptosis, immune function, and fibrogenesis [20,21]. Decreased levels of glutathione are also associated with other psychiatric conditions including bipolar disorder, major depressive disorder, and schizophrenia [22]. Glutathione exists primarily in its thiol reduced form (GSH) accounting for >98% of total glutathione versus its disulfide-oxidized form (GSSG) [20]. The antioxidant function of glutathione is mediated by glutathione peroxidase, which reduces ROS such as hydrogen peroxide and lipid peroxide, resulting in GSSG. GSH is regenerated via GSSG reductase and NADPH for further antioxidative action (Figure 1). The synthesis of glutathione is primarily limited by the rate-limiting agent, cysteine. Cysteine can be derived in several ways including diet, protein breakdown and recycling in the liver, the transsulfuration of methionine (Figure 2) [20]. Another important molecule is N-acetylcysteine (NAC), which is an acetylated cysteine residue that can serve as a glutamatergic modulator, a free radical scavenger, and as a cysteine donor to maintain GSH status [23].

NAC has been a target of interest primarily for its antioxidative role in a variety of disorders including neural cell survival, cell signaling, neurodegenerative diseases, multiple sclerosis, traumatic brain injuries, and other psychotic disorders [24,25]. Its antioxidant effects can be extrapolated to play a role in the redox balance in ASD. There have been recent placebo-controlled pilot studies that have looked at NAC in ASD patients directed toward the treatment of irritability, aggression, self-injurious behavior and tantrums [26,27]. Harden et al. found a significant reduction in irritability symptoms that were measured by the Aberrant Behavior Checklist in a placebo-controlled pilot trial of NAC in children with ASD [28]. Another 12-week, randomized, double-blind, placebo-controlled trial of oral NAC in children with ASD found NAC to be safe. It was not noted to have a significant impact on core social impairment seen in ASD, but the authors did indicate that larger scaled trials are needed in order to predict a good treatment response [26]. A meta-analysis by Lee et al. found that NAC supplementation alleviated hyperactivity and irritability in ASD, based on the Aberrant Behavior Checklist. More importantly, the study demonstrated the safety and tolerability of NAC supplementation in children with ASD [29].

Further research into anti-oxidative agents for ASD begs the question of whether direct supplementation with glutathione is equally or more efficacious than NAC supplementation due to bypassing the rate-limiting transsulfuration step (cysteine) in glutathione synthesis. Despite its proposed benefits, oral glutathione supplementation failed to show changes to the observed biomarkers of oxidative stress and glutathione status, due to the enzymatic degradation of supplemental glutathione in the intestine [30]. The issue of poor bioavailability was recently circumvented with the development and introduction of a novel glutathione that aids in gastrointestinal uptake [31]. One such formulation, termed Opitac^TM^ glutathione, is derived from the fermentation of Torula yeast. When administered at 50 mg/kg body weight, the level of Opitac^TM^ glutathione in the protein-bound fraction of plasma significantly increased 60–120 min after supplementation and the level of glutathione and related compounds (γGlu-Cys and Cys-Gly) were sustained for at least 2 h [32]. Other studies on the oral administration of glutathione also show significantly elevated body stores of glutathione in addition to improved markers of immune function [31,33]. This open-label pilot case series examines the use of Opitac^TM^ glutathione in the symptomatic treatment of ASD in children.

## 2. Methods and Materials

### 2.1. Study Design

This 12-week open-label pilot study was conducted to investigate the efficacy of glutathione use as a supplement for the symptomatic treatment of irritability and aggression in children with ASD, as defined by criteria set forth in the DSM-5. Trials were conducted in the outpatient setting at the University of Chicago. Institutional review board proposal was submitted and approved for the intent and purpose of this experimental study. The study is registered under NCT05954052 with clincialtrials.gov. All studied subjects agree to this voluntary study without financial incentives or otherwise. After obtaining informed consent from parents, subjects are screened for inclusion and exclusion criteria as highlighted below. For the purpose of this study, oral glutathione refers to oral supplementation with Opitac^TM^ glutathione.

### 2.2. Inclusion and Exclusion Criteria

Inclusion criteria included the following: (a) children and adolescents of both sexes between ages 4–17; (b) diagnosis of ASD as determined by criteria set forth in DSM-5 (Appendix A); (c) parent(s) and guardian(s) of children ages 4–17 with a current diagnosis of ASD who agree to accompany the patient for initial evaluation and consistent outpatient follow up appointments through to the end of the study period; (d) patient is clinically stable with or without medications and interventions for a period of at least 2 weeks prior to study enrollment; (e) patient will not undergo any changes to current medical and psychosocial intervention(s) during planned treatment period.

Exclusion criteria included the following: (a) unstable medical illness or clinically significant abnormalities on physical examination; (b) history of seizures; (c) history of hematological disorders such as anemia, coagulopathies, neoplasia; (d) history of myocardial infarction within the last 6 months; (e) current pregnancy, lactation, or inadequate contraception in females of childbearing potential; (f) current or recent (past 3 months) substance use or dependence; (g) illegal substance use within 2 weeks of study initiation; (h) previous treatment with glutathione; (i) current treatment with supplements that interfere with glutathione levels including NAC, milk thistle, vitamin C, vitamin B, grape seed extract, amino acids, or zinc; (j) current treatment with medications with known glutamatergic properties such as dextromethorphan, d-cyloserine, amantadine, memantine, lamotrigine, or riluzole; (k) underlying asthma that may potentiate worsening of respiratory symptoms due to glutathione use.

### 2.3. Interventions

Subjects were followed over a 12-week course with an initial intake appointment for baseline screening of vital signs, oxidative laboratory work, Social Responsiveness Scale, Aberrant Behavior Checklist, and Clinical Global Impression scale. The use of oral glutathione was discussed with subjects and their parents who provided signed consent for this trial.

Opitac^TM^ glutathione oral capsules (Jarrow Formulas) were the only medication that was dispensed to subjects for the duration of the study. The proposed dose range for glutathione in this study was 1000 mg–3000 mg/day based on the subject’s weight. A previous observational study of children with cystic fibrosis showed that this population responded well and were able to absorb glutathione with a daily dose of 65 mg/kg [34]. Given that cystic fibrosis is a condition that typically results in malabsorption of fats and other products, it is inferred that subjects with normal gastrointestinal (GI) systems may absorb glutathione effectively at a slightly lower starting dose, proposed to be ~32.5 mg/kg. No adverse events were noted in the above-mentioned study; however, to ensure tolerability in our subjects, a conservative approach was selected to ensure safety by starting at a low dose and titrating up. The dosing regimen selected is based on subject weight and is divided as follows: (a) for subjects 40 kg or less: 500 mg by mouth twice a day for two weeks, then 1000 mg by mouth twice a day for ten weeks; (b) for subjects greater than 40 kg: 500 mg by mouth twice a day for two weeks, then 1000 mg by mouth twice a day for two weeks, followed by 1000 mg by mouth each morning and 2000 mg by mouth every afternoon for eight weeks. If subjects do not tolerate the dose increase at the start of week 3, they are kept at the last tolerated dose and maintained for the remainder of the trial.

Subjects were followed up using several qualitative measures including the Aberrant Behavior Checklist (ABC), Social Responsiveness Scale (SRS), Clinical Global Impression Scale—Severity (CGI-S), and Side Effects Checklist (SEC) (Appendix B) through course of study.

### 2.4. Outcome Measures

Our primary outcome was to monitor therapeutic efficacy of glutathione in treating symptoms of ASD in children. Our secondary outcome was to evaluate the tolerability of oral glutathione in children with ASD. The following scales and checklists were used for the goals of the study.

#### 2.4.1. Oxidative Labs

Subjects underwent laboratory studies to assess oxidative burden pre- and post-treatment during the study period. Oxidative Stress Analysis 2.0 was performed by Geneva Diagnostics and included a battery of tests to quantify oxidative stress. Specifically, this panel looks at (I) reduction–oxidation reserve including glutathione, total antioxidant capacity, cysteine, cystine, cysteine/cystine ratio, sulphate, cysteine/sulphate ratio; (II) protective enzymes including superoxide dismutase and glutathione peroxidase; (III) cellular damage as measured by lipid peroxides.

#### 2.4.2. Aberrant Behavior Checklist (ABC)

This is 58-item symptom checklist that assesses behavioral problems in adults and children with developmental disabilities including ASD, intellectual disability, epilepsy, etc. The checklist is a versatile assessment tool that can be used in a variety of settings including home, community settings, educational settings, and in developmental centers. The checklist can also be administered by various direct caregivers including but not limited to parents, healthcare workers, and educators. Items are divided into 5 domains or subscales that assess (I) irritability and agitation; (II) lethargy and social withdrawal; (III) stereotypic behavior; (IV) hyperactivity and non-compliance; (V) inappropriate speech. Subscale scores at baseline are compared to post-treatment scores to assess primary and secondary outcomes.

#### 2.4.3. Social Responsiveness Scale (SRS)

This scale is a respondent-based outcome measure used to assess deficits and symptoms related to ASD. The scale comes in two forms differentiated by gender and age. The scale can be administered by parents/caregivers and consists of subscales that assess (I) social awareness; (II) social cognition; (III) social communication; (IV) social motivation; (V) autism mannerisms. The raw subtotal of each domain is converted to a standardized score (T-score) and summed to calculate a total SRS score. All T-scores have a mean of 50 points and a standard deviation of 10 points. Clinical severity of ASD as determined by SRS total t-score is as follows: (I) less than or equal to 59 represents low-to-no symptom impact (normal); (II) between 60–65 represents mild-to-moderate deficits in social interaction; (III) between 66–75 represents moderate deficit in social interaction; (IV) greater than or equal to 76 represents severe (strongly associated with a clinical diagnosis of ASD). Repeated SRS assessments are compared and monitored to track progress toward achieving treatment plan goals.

#### 2.4.4. Clinical Global Impressions (CGI) Scale

This brief, 3-item assessment tool provides a global rating of illness severity (CGI-S), global improvement or change (CGI-I), and therapeutic efficacy (CGI-E). CGI-S is measured on a 7-point scale ranging from 1 (normal, not at all ill) to 7 (among the most extremely ill patients). CGI-I is measured on a 7-point scale as well, ranging from 1 (very much improved) to 7 (very much worse). CGI-E takes into account both the therapeutic effect and adverse events, ranging from 1 (marked therapeutic effect) to 04 (unchanged or worse). Our study utilizes a CGI-E final index score ranging from 0–16 which depends on separate subscale ratings of therapeutic benefits versus side effects.

#### 2.4.5. Side Effect Checklist (SEC)

This in-house glutathione side effect questionnaire (Appendix B) was developed to assess for common medication side effects including GI upset, dry mouth, headache, and allergic symptoms. SEC also assessed for duration of adverse side effects and frequency of events. Any other adverse effects observed that were not otherwise asked were specified under ‘others’ on the questionnaire.

### 2.5. Statistical Analyses

The CGI severity scale was used to assess clinically significant change after treatment. For someone to have a clinically significant change, her or his final CGI severity score had to be less than the cutoff score, and his or her change from baseline had to be greater than the reliable change index. The statistical significance of pre- and post-treatment ABC scores were determined via paired *t*-test with *p*-value set to <0.05.

## 3. Results

### 3.1. Study Population

After screening the potential subjects for the inclusion and exclusion criteria, this pilot study recruited a total of six subjects, all of whom met DSM-5 criteria for a diagnosis of autism spectrum disorder. Parental consent was obtained for each participant during enrollment prior to the study. The demographics of the study group are outlined in Table 1. The mean age of the participants in the study group was 14.7 years.

### 3.2. Oxidative Stress Analysis

The participants all underwent baseline oxidative stress screening prior to glutathione treatment and post-glutathione treatment upon completion of the study where appropriate. One patient was unable to complete the full study and did not undergo the post-treatment oxidative stress screen. The results of the oxidative stress testing are outlined in Table 2.

Three subjects’ baseline glutathione levels were below the normal ranges during the pre-treatment assessment. Two of these three subjects’ below normal glutathione levels improved to normal ranges with oral glutathione supplementation. The total antioxidant capacity did not improve in one subject, and even worsened in another subject. Cysteine is the rate-limiting amino acid for glutathione production. There were no pre- and post-treatment differences in the cysteine levels in all patients supplemented with oral glutathione. Sulfate is produced from cysteine via sulfoxidation. Sulfate is important in detoxification reactions, and is essential for GI function and joint health. A high cysteine/sulfate ratio or low sulfate level may indicate oxidative stress. Two subjects showed greater than one standard deviation improvement in sulfate levels after treatment, and three subjects showed at least one standard deviation decrease in their cysteine/sulfate ratio after treatment. Cystine is the oxidized product of cysteine and is the predominant form in blood due to its greater relative stability. Both the cystine and cysteine/cystine ratios were within the normal ranges for all patients. The oxidative panel looked at two protective enzymes, namely, superoxide dismutase and glutathione peroxidase. The function of these enzymes is to protect against oxidative stress by reducing superoxide anions to hydrogen peroxide and from hydrogen peroxide to water, respectively. Four of the six subjects experienced >1 std deviation increase in glutathione peroxidase from baseline, and two of the six patients experienced >1 std deviation increase in superoxide dismutase from baseline. One patient with baseline >2 standard deviations below the normal ranges of glutathione peroxidase and superoxide dismutase did not experience any significant enzyme level changes with glutathione supplementation. Lipid peroxides are a direct indicator of oxidative damage to polyunsaturated fatty acids, suggesting an imbalance of redox reactions favoring oxidation. Lipid peroxides were unremarkable for most subjects. Of note, the patient who dropped out had low baseline levels of glutathione and an elevated baseline level of the cysteine/sulfate ratio, superoxide dismutase, and lipid peroxides.

### 3.3. Behavioral Outcomes

The ASD severity was measured using SRS. All subjects underwent SRS screening during the initial enrollment, with the results outlined in Table 3. In terms of the baseline ASD severity, one subject scored within the normal/not severe range, one subject scored in the moderate severity range, and four subjects scored within the severe range. Of the five domains assessed in the SRS, social communication and autism mannerisms were identified as the most problematic and contributed the most to the overall SRS total severity score.

ABC was another scale used to track the ASD treatment progress with oral glutathione supplementation. The pre- and post-treatment subscores in five different domains are outlined in Table 4. The statistical analyses of these subscores are shown in Table 5. The most prominent and affected domains in the study subjects are the symptoms of hyperactivity followed by irritability. The two domains of lesser concern are inappropriate speech followed by stereotypy. In terms of the greatest absolute change in any subscale, hyperactivity improved the most between pre- and post-treatment outcomes. None of the mean differences between the pre- and post-treatment subscores in any domain reached statistical significance.

### 3.4. Safety Evaluation

Oral glutathione supplementation was generally well-tolerated throughout the course of the study. There were minimal adverse effects reported, as summarized in Table 6. Upset stomach (*n* = 4) was the most reported side effect. One subject reported dry mouth, and one subject reported skin flushing. The parents utilized the write-in option (“others”) on the SEC to report the symptoms of constipation, increased hyperactivity, increased irritability, and increased aggression. Only one subject dropped out from the study due to a significant increase in irritability.

The CGI scale was also used as a tool to measure the improvement with oral glutathione supplementation; the results are summarized in Table 7. The pre-treatment CGI-S was rated from 2 (borderline ill) to 5 (markedly ill). One subject went from a pre-treatment CGI-S of 4 (moderately ill) to a post-treatment CGI-S of 5 (markedly ill). All other subjects did not experience a change in the CGI-S pre- and post-treatment. In assessing the CGI-I, one subject scored a 4 (no change/improvement), four subjects scored a 3 (minimally improved), and one subject scored a 6 (much worse). CGI-E is divided into two components: therapeutic efficacy and side effects. In terms of the therapeutic efficacy, two patients experienced no changes with treatment and four subjects were rated to have minimal therapeutic efficacy. In terms of side effects, five subjects experienced no side effects, while the one subject who dropped out of the study was rated to experience significant side effects.

## 4. Discussion

Our pilot investigation examined the utility and tolerability of oral supplementation with glutathione as a treatment for patients with ASD. A similar study by Kern et al. looked at glutathione supplementation for the treatment of ASD. Kern et al. demonstrated the safety profile of oral glutathione supplementation and significant increases in plasma reduced glutathione using glutathione dosages significantly lower (ranging from 50–200 mg/30 lbs or 3.67–14.7 mg/kg) than those used in this pilot study; however, they did not demonstrate a statistically significant difference in the increased whole blood glutathione. As previously mentioned in the study design, patients with cystic fibrosis require ~65 mg/kg of glutathione for adequate absorption. Our study utilizes a more conservative approach of ~32.5 mg/kg of glutathione, which is still well above the range used by Kern et al. Even at higher glutathione doses, our study once again demonstrates a good safety profile for glutathione use.

The study by Kern et al. also showed statistically significant increases in plasma transsulfuration metabolites including sulfate, cysteine, and taurine following glutathione supplementation, but they were unable to delineate whether the increased metabolites were truly due to glutathione’s effect on the transsulfuration pathway or whether the increases in transsfulfuration metabolites were due to the increased breakdown of glutathione [35]. Our case series redemonstrates an increase in transsulfuration metabolites with oral glutathione supplementation, but the changes are not statistically significant. Supplementation with oral glutathione may improve oxidative markers but does not necessarily translate to the observed clinical improvement of subjects with ASD.

Glutathione was generally well-tolerated except in the case of one subject, who experienced a significant increase in irritability and ultimately discontinued their participation in the study. Stomach upset was reported in four of the six subjects in the study as a side effect with oral glutathione treatment. Stomach upset and GI side effects may not necessarily be attributed to oral glutathione alone. With the exception of irritability, all other reported side effects with oral glutathione (Table 6) did not persist and did not serve as a limitation to continuing treatment. Studies have shown that the prevalence of patients with ASD and associated gastrointestinal dysfunction ranges from 9–91% [36,37]. Children with ASD and anxiety seem to have greater risk for lower GI issues, suggesting an exaggerated stress response. Additionally, it is suggested that a subset of children with ASD have automatic nervous system dysfunction leading to greater GI instability [37]. The GI symptoms of abdominal pain, gas, diarrhea, and constipation are associated with a greater risk for worsened ASD symptoms including withdrawal, irritability, and hyperactivity [38]. Recent studies have demonstrated that the dysbiosis of gut microbiota can lead to distinct autistic phenotypes ranging from mild irritability to extreme behavioral concerns in ASD [37,39]. Certain bacteria and viruses can alter gut microbiome by producing ROS and triggering inflammatory pathways. Higher levels of nitrites among other biomarkers such as short-chain fatty acids, lipopolysaccharides, beta-cresol, and bacterial toxins in children with ASD supports the theory that ASD is a brain–gut–microbiome disorder [39,40]. There is a major research focus on protecting the gut microbiome as a treatment for ASD.

The baseline oxidative stress analysis was crucial in understanding the redox imbalance in the patients with ASD. In the case of the subject that dropped out of the study, the pre-treatment laboratory work shows a baseline depletion of the glutathione reserve, high levels of superoxide dismutase despite normal glutathione peroxidase, and elevated lipid peroxides. Another subject shows baseline low levels of antioxidant capacity, significantly elevated glutathione peroxidase, and significantly elevated superoxide dismutase. Both subjects had high oxidative burdens prior to treatment and did not seem to experience any clinical or redox benefit with oral glutathione supplementation. A possible explanation for this phenomenon is that these subjects are at maximum physiologic compensation with a high baseline oxidative burden. The further addition of glutathione is unable to surpass the physiologic cap on the redox balance. The results may indicate that subjects with high baseline oxidative burden may be poor responders to oral glutathione supplementation. All other subjects showed improved total antioxidant capacity, increased sulfate levels, and significant increases in glutathione peroxidase and superoxide dismutase. Another observation is that oxidative burden does not necessarily translate to clinical severity, as indicated via the CGI scores.

The clinical progress in this study was monitored via ABC. There were decreases in the post-treatment mean scores across all ABC domains as compared to the pre-treatment scores, but the mean differences were not statistically significant. However, it is important to note that despite the lack of statistical significance, there was a mild improvement in the severity of ASD symptoms in 66.7% of the patients, according to the CGI-I. This translates to an observable clinical significance and improvement in the quality of life of some patients with ASD.

As referenced, an imbalance in redox reactions is only one of the many factors that contribute to ASD. Impaired neurotransmission, immune dysregulation in the brain, mitochondrial dysfunction, and a mix of environmental factors may also contribute to treatment response. A recent study by Huang et al. demonstrates the critical role of glutathione in astrocytes to help maintain blood–brain barrier stability by suppressing endothelial cell tight junction phosphorylation and delocalization [41]. The maintenance of blood–brain barrier homeostasis prevents the intrusion of ROS, neurotoxic debris, and inflammatory cytokines from disrupting the brain parenchyma.

Given the association between ASD and gastrointestinal abnormalities, food picking, and food aversion, many individuals with ASD have concurrent deficiencies in macro and micronutrients. The most well-studied agents are vitamin A, vitamin B_1_ (thiamine), vitamin B_6_ (pyridoxine), and vitamin B_12_ (cobalamin). Vitamin B_1_ plays a role in multiple systems including the regulation of apoptosis in response to oxidative stress [19]. Vitamin B_1_ deficiency is associated with delayed language development in childhood [42]. Vitamin A plays a role in multiple systems including the regulation of apoptosis, neurogenesis, immune health, and serotonin systems [19]. Supplementation with vitamin A has been shown to have a positive effect on social memory, communication, and coordination [43]. Vitamin B_6_ is an important coenzyme required for the general metabolic maintenance of cells including the degradation of amino acids and the synthesis of many neurotransmitters including dopamine, GABA, serotonin, noradrenaline, and histamine, among others [19]. Supplementation with concurrent vitamin B_6_ with Mg^2+^ (cofactor) has shown improvements in social interaction and restrictive behaviors [44]. Vitamin B_12_ is an important cofactor in the methionine cycle, which ultimately generates cysteine to be consumed in the transsulfuration pathway to produce reduced GSH. Simply put, vitamin B_12_ plays a crucial role in maintaining the redox balance in the body by generating antioxidant species. Supplementation with subcutaneous vitamin B_12_ injections has been shown to improve sleep, gastrointestinal symptoms, hyperactivity, and non-verbal intellectual quotient [45]. Other nutrients such as vitamin B_7_, vitamin D, zinc, omega fatty acids, sulforaphane, and chelating agents have also been research targets for the symptomatic management of ASD; no single treatment has been shown to be effective as a monotherapy [45,46,47]. Oral glutathione may work synergistically with other nutritional supplementation to generate a greater effect size in treating ASD.

Another key component in the baseline redox balance involves genetics. Glutathione S-transferases (GST) are a family of enzymes that aid in the conjugation of reduced GSH to xenobiotics for detoxification and also helps reduce endogenous oxidative species. All four major classes of GST have been shown to exhibit genetic polymorphisms reducing their antioxidative ability and are associated with psychiatric pathologies such as ASD [48,49]. Common GST polymorphisms include GSTA1, GSTM1, GSTT1, and GSTP1. Some protective polymorphisms include the GSTA1*CC genotype, which has predicted lower non-verbal communication; the GSTP1*allele genotype, which is associated with better cognitive functioning in ASD; and the GSTM1-active genotype, which has predicted higher adaptive functioning [50]. Furthermore, the GSTM1-active genotype contributes to the overall antioxidant capacity and is moderated by external factors such as maternal smoking during pregnancy [50]. The null phenotypes and moderated phenotypes lack the protective benefit against ASD and other psychiatric illnesses. It is important to keep in mind that ASD is a complex developmental disorder. Understanding the interaction between genetic predisposition and environmental risks may help mitigate ASD impairment to improve cognitive functioning and behaviors.

The method of delivery is an important consideration in this study. Opitac^TM^ glutathione was used as a way to increase bioavailability, with better central nervous system (CNS) penetration. There is a growing body of research that takes this idea one step further to explore the tolerability and efficacy of intranasal glutathione. Intranasal administration bypasses the blood–brain barrier, which filters out 98–100% of lipophilic molecules. Although intranasal glutathione has not been studied for ASD, treatment with intranasal glutathione has been studied for Parkinson’s disease and shows an excellent penetration rate and increased levels of GSH in brain tissue [51]. Newer advances in bioengineering utilize nasal permeability enhancers, gelling agents, and nanoparticle formulations that optimize drug delivery into brain tissue [52]. Other considerations for parenteral glutathione administration for bioavailability include the intravenous route. There is no current data on the use of IV glutathione in the treatment of ASD, but IV glutathione has been used for Parkinson’s disease and as a skin lightening therapy in the literature [53,54,55]. Current studies on IV glutathione have failed to evaluate the long-term safety profile of its use [55]. Additionally, it may be of limited clinical benefit in the ASD population based on behavioral limitations to adhere with continuous treatment. Numerous studies have explored the use of NAC in the context of ASD, and this represents the second study focusing on the application of glutathione. To gain a more comprehensive understanding of the redox system’s involvement in ASD, it is essential that future research endeavors include comparative studies between NAC and glutathione in the context of this disorder. Such investigations will shed light on the relative efficacy and mechanism of redox and its role in ASD. Utilizing biomarkers and newly acquired genetic profiles for autism, along with insights into the redox systems, can pave the way for future studies to embrace precision medicine and personalized interventions in heterogeneous groups and phenotypes.

## 5. Limitations

This case series was limited by its small sample size, which served to impact the power of the study. None of the observed changes in the pre- and post-treatment oxidative laboratory markers and ABC scores were statistically significant. To expand on this limitation, one subject dropped out of the study and was unable to provide post-treatment scores at the end of the intended study period. The observations with this subject were based on the intent-to-treat analysis, with the last observation carried forward. Another limitation to the study is the use of subjective and informant-based instruments (SRS, ABC, SEC, CGI) due to the lack of reliable and readily available objective or performance-based instruments to measure the clinical severity of ASD and treatment outcomes. Resource restrictions also limited potential data collection, as we would have preferred a more frequent oxidative stress panel, ABC, SRS, and other scales to track the treatment progress.

Despite these limitations, this pilot study is a proof of concept that further studies are warranted with greater power to look at the therapeutic potential of oral glutathione supplementation to mitigate the presenting symptoms in ASD. The study also demonstrates a decent safety profile regarding oral glutathione supplementation. Future research should aim to replicate this study with a well-characterized population of ASD subjects to further assess glutathione’s key role as a master antioxidant in treating the redox imbalance observed in ASD.

## Figures and Tables

**Table 1:** Patient demographics for study sample.

	Patient 1	Patient 2	Patient 3	Patient 4	Patient 5	Patient 6
Demographics						
Sex	M	M	F	F	M	M
Age at presentation	16	15	18	15	14	10
Race	Hispanic	Caucasian	African American	African American	Caucasian	Hispanic

**Table 2:** Comparison of baseline (pre) and post-treatment (post) oxidative lab values.

	Patient 1		Patient 2		Patient 3		Patient 4		Patient 5		Patient 6		Reference
	Pre	Post	Pre	Post	Pre	Post	Pre	Post	Pre	Post	Pre	Post	
Glutathione	928	1067	567	1060	1090	1037	1333	1141	787	-	1115	825	>=669 micromol/L
Total Antioxidant Capacity	0.64	0.7	0.56	0.49	0.39	0.48	0.61	0.79	0.82	-	-	0.66	>=0.54 mmol/L
Cysteine	0.75	0.67	0.87	0.59	0.79	0.85	0.75	0.82	0.84	-	-	0.66	0.46–1.20 mg/dL
Sulfate	4.2	4.7	4.5	5.9	3.3	4.2	4.6	6.2	5.7	-	-	4.8	3.0–5.9 mg/dL
Cysteine/Sulfate	0.18	0.14	0.19	0.1	0.24	0.2	0.16	0.13	0.15	-	-	0.14	0.12–0.32
Cystine	2.09	2.36	2.52	2.18	2.53	2.43	2.34	2.51	2.33	-	-	2.4	1.60–3.20 mg/dL
Cysteine/Cystine Ratio	0.36	0.28	0.35	0.27	0.31	0.35	0.32	0.33	0.36	-	-	0.28	0.17–0.50
Glutathione Peroxidase	26.2	33.3	21.7	37.2	40.9	51.9	32.8	40.4	26.3	-	35.6	47.5	20.0–38.0 U/g Hb
Superoxide Dismutase	12,576	20,038	14,207	19,121	20,697	19,330	11,393	12,112	18,992	-	7224	12,655	5275–16,662 U/g Hb
Lipid Peroxides	5.7	6.1	5.5	6.7	4.3	2.1	6.1	3.7	7.1	-	-	3.1	<=10.0 micromol/L
	>2 standard deviations below mean												
	Between 1–2 SD below mean												
	Within 1 standard deviation from mean												
	Between 1–2 SD above mean												
	>2 standard deviations above mean												

**Table 3:** Baseline Social Responsive Scale (SRS).

Social Responsiveness Scale (SRS)	Patient 1	Patient 2	Patient 3	Patient 4	Patient 5	Patient 6
SRS Social Awareness	65	86	56	87	51	>90
SRS Social Cognition	83	87	77	>90	46	>90
SRS Social Communication	>90	>90	56	>90	49	>90
SRS Social Motivation	81	>90	73	84	38	83
SRS Autism Mannerisms	89	89	74	>90	68	>90
SRS Total (Severity)	88 (Severe)	>90 (Severe)	68 (Moderate)	>90 (Severe)	51 (Normal)	>90 (Severe)

**Table 4:** Comparison of Aberrant Behavior Checklist (ABC) at baseline and post-treatment.

	Patient 1		Patient 2		Patient 3		Patient 4		Patient 5		Patient 6	
Aberrant Behavior Checklist (ABC)	Baseline	Post Treatment	Baseline	Post Treatment	Baseline	Post Treatment	Baseline	Post Treatment	Baseline	Post Treatment	Baseline	Post Treatment
ABC Irritability	28	21	25	23	3	10	38	23	11	18	35	21
ABC Lethargy	25	20	29	20	9	13	26	19	2	0	21	10
ABC Stereotypy	13	9	9	11	9	12	15	11	4	1	8	3
ABC Hyperactivity	24	17	33	20	9	15	42	34	14	11	40	11
ABC Inappropriate Speech	10	6	6	8	5	9	7	8	5	1	11	5

**Table 5:** Mean difference and clinical significance of Aberrant Behavior Checklist (ABC) pre- and post-treatment scores.

	Mean (SD)		Mean Difference (CI)	*p*-Value
Aberrant Behavior Checklist (ABC)	Pre Treatment	Post Treatment		
ABC Irritability	23.33 (13.72)	19.33 (4.93)	4.0 [−6.24, 14.24]	0.3614
ABC Lethargy	18.67 (10.75)	13.67 (7.87)	5.0 [−0.67, 10.67]	0.0728
ABC Stereotypy	9.67 (3.88)	7.83 (4.67)	1.83 [−1.77, 5.43]	0.2474
ABC Hyperactivity	27.0 (13.65)	18.0 (8.58)	9.0 [−3.26, 21.26]	0.1177
ABC Inappropriate Speech	7.33 (2.58)	6.17 (2.93)	1.17 [−3.05, 5.39]	0.5090

**Table 6:** Side effects of glutathione during course of treatment.

Side Effects	Number of Patients (n = 6)
Upset Stomach	4
Diarrhea	0
Dizziness	0
Dry Mouth	1
Headache	0
Skin Rash	0
Skin Flushing	1
Difficulty Breathing	0
Chest Tightness	0
Other:	
Constipation	1
Increased Hyperactivity	1
Increased Irritability	1
Increased Aggression	1

**Table 7:** Comparison of pre- and post-treatment CGI scores. Note: TE, therapeutic effect; SE, side effects.

	Patient 1	Patient 2	Patient 3	Patient 4	Patient 5	Patient 6
Pre-Treatment CGI						
CGI Severity	2 (Borderline ill)	4 (Moderately Ill)	5 (Markedly Ill)	4 (Moderately Ill)	3 (Mildly Ill)	5 (Markedly Ill)
Post Treatment CGI						
CGI Severity	2 (Borderline ill)	4 (Moderately Ill)	5 (Markedly Ill)	5 (Markedly Ill)	3 (Mildly Ill)	5 (Markedly Ill)
CGI Improvement	3 (Minimally Improved)	3 (Minimally Improved)	3 (Minimally Improved)	3 (Minimally Improved)	6 (Much Worse)	4 (No Change)
Efficacy Index	9 (Minimal TE, No SE)	9 (Minimal TE, No SE)	9 (Minimal TE, No SE)	9 (Minimal TE, No SE)	15 (Unchanged TE, Significant SE)	13 (Unchanged TE, No SE)

**Figure A1:**
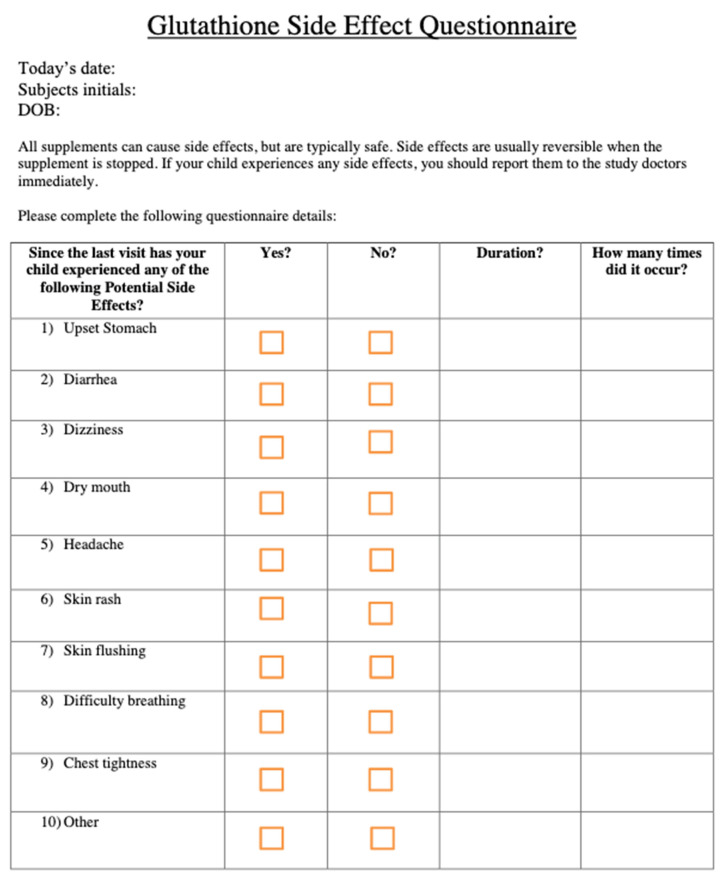
Glutathione Side Effect Questionnaire.

## Data Availability

The study is registered with the clincialtrials.gov.

## Appendix A. Diagnostic Criteria

The following diagnostic criteria for ASD will be utilized. The criteria are defined by the Diagnostic and Statistical Manual for Mental Disorders (DSM-5) for a diagnosis of autism spectrum disorder:

A. Persistent deficits in social communication and social interaction across multiple contexts, as manifested by the following, currently or by history (examples are illustrative, not exhaustive, see text):
  1. Deficits in social-emotional reciprocity, ranging, for example, from abnormal social approach and failure of normal back-and-forth conversation; to reduced sharing of interests, emotions, or affect; to failure to initiate or respond to social interactions.
  2. Deficits in nonverbal communicative behaviors used for social interaction, ranging, for example, from poorly integrated verbal and nonverbal communication; to abnormalities in eye contact and body language or deficits in understanding and use of gestures; to a total lack of facial expressions and nonverbal communication.
  3. Deficits in developing, maintaining, and understanding relationships, ranging, for example, from difficulties adjusting behavior to suit various social contexts; to difficulties in sharing imaginative play or in making friends; to absence of interest in peers.

Specify current severity: Severity is based on social communication impairments and restricted repetitive patterns of behavior.

B. Restricted, repetitive patterns of behavior, interests, or activities, as manifested by at least two of the following, currently or by history (examples are illustrative, not exhaustive; see text):
  1. Stereotyped or repetitive motor movements, use of objects, or speech (e.g., simple motor stereotypies, lining up toys or flipping objects, echolalia, idiosyncratic phrases).
  2. Insistence on sameness, inflexible adherence to routines, or ritualized patterns or verbal nonverbal behavior (e.g., extreme distress at small changes, difficulties with transitions, rigid thinking patterns, greeting rituals, need to take same route or eat food every day).
  3. Highly restricted, fixated interests that are abnormal in intensity or focus (e.g., strong attachment to or preoccupation with unusual objects, excessively circumscribed or perseverative interest).
  4. Hyper- or hyporeactivity to sensory input or unusual interests in sensory aspects of the environment (e.g., apparent indifference to pain/temperature, adverse response to specific sounds or textures, excessive smelling or touching of objects, visual fascination with lights or movement).

Specify current severity: Severity is based on social communication impairments and restricted, repetitive patterns of behavior.

C. Symptoms must be present in the early developmental period (but may not become fully manifest until social demands exceed limited capacities or may be masked by learned strategies in later life).
D. Symptoms cause clinically significant impairment in social, occupational, or other important areas of current functioning.
E. These disturbances are not better explained by intellectual disability (intellectual developmental disorder) or global developmental delay. Intellectual disability and autism spectrum disorder frequently co-occur; to make comorbid diagnoses of autism spectrum disorder and intellectual disability, social communication should be below that expected for general developmental level.

Note: Individuals with a well-established DSM-IV diagnosis of autistic disorder, Asperger’s disorder, or pervasive developmental disorder not otherwise specified should be given the diagnosis of autism spectrum disorder. Individuals who have marked deficits in social communication, but whose symptoms do not otherwise meet criteria for autism spectrum disorder, should be evaluated for social (pragmatic) communication disorder.

Specify if:

- With or without accompanying intellectual impairment
- With or without accompanying language impairment
- Associated with a known medical or genetic condition or environmental factor
- Associated with another neurodevelopmental, mental, or behavioral disorder
- With catatonia
